# QuickStats

**Published:** 2014-03-07

**Authors:** FROM THE NATIONAL CENTER FOR HEALTH STATISTICS

**Figure:**
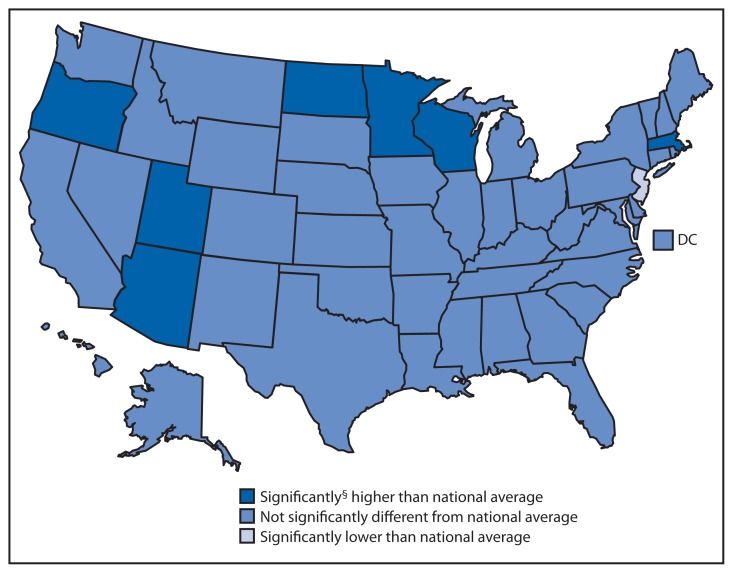
Percentage of Office-Based Physicians Using an Electronic Health Record (EHR) System,^*^ by State — National Ambulatory Medical Care Survey,^†^ United States, 2013 ^*^ An EHR system is a medical or health record system that is entirely or partially electronic. ^†^ A sample survey of office-based physicians. ^§^ All differences have been tested and determined to be statistically significant, unless otherwise stated.

In 2013, the percentage of physicians using an EHR system was higher than the national average (78%) in seven states (Arizona, Massachusetts, Minnesota, North Dakota, Oregon, Utah, and Wisconsin) (range = 87%–94%) and was lower than the national average in New Jersey (66%).

**Source:** NCHS Research Data Center: what’s new? Winter 2014. National Electronic Health Records Survey (NEHRS). Survey data available through the NCHS Research Data Center at http://www.cdc.gov/rdc/leftbrch/whatnew.htm.

**Reported by:** Esther Hing, MPH, ehing@cdc.gov, 301-458-4271; Chun-Ju Hsiao, PhD.

